# Supplementary material for: A comparative analysis of objectively assessed physical activity levels in kindergarten and home among children aged 5 to 6
Source: PLoS One. 2026 May 15;21(5):e0349582. doi: 10.1371/journal.pone.0349582 (PMC13178922; doi:10.1371/journal.pone.0349582)
Supplement: Attachment — Submitted filename: Study review 12_8.docx [file pone.0349582.s001.docx]

Introduction p2

In this section there are numerous compound sentences where two ideas are joined with “and”. A compound sentence should not address 2 disparate ideas. Most paragraphs lack a thesis statement so then the paragraph is a collection of ideas instead of presenting a cohesive idea.

Re-phrase the opening sentence to begin with something other than “there”. One way to write this sentence would be: Adequate levels of physical activity for school-age and preschool children improves……

The second paragraph beginning on p2 does not have a thesis statement. Multiple areas of focus are presented in the paragraph. The paragraph is not cohesive.

P3 – The paragraph beginning with “The level of PA” is not a paragraph because it only contains 2 sentences.

“A sedentary lifestyle” is not a paragraph.

“Over the past two decades, research in Poland has predominantly relied on subjective assessments of PA, employing diagnostic survey methods [36].” – poor sentence structure. A better option would be “Over the past two decades in Poland, multiple research projects utilizing subjective assessments of PA have been conducted.”

Combine these two paragraphs and use a thesis statement. I would recommend introducing this earlier into the Introduction since you are correlating

Tables – add in the type of statistical analysis to help the reader better understand how you arrived at the conclusions.

Results – add in the type of statistical analysis to help the reader better understand the results.

Discussion

This needs to be in your Introduction “A meta-analysis combining accelerometer-measured MVPA estimates from 29 studies involving over 6,000 preschool-age children found that kindergarteners spend only about 5.5% of their time on MVPA per day [40]. Similarly, in subsequent studies, Van Cauwenberghe et al. [41] on a sample of 1004 Australian kindergarteners showed that the MVPA rate (hour-by-hour 8 percentage analysis) averaged 4% in kindergarten and 5% at home. Brown et al. [42] reported that children spent 3% on MVPA in kindergarten. Slightly better outcomes were observed by Pate et al. [43], they used Actigraph for two weeks and showed that kindergarteners spent 13% on MVPA. In one recent study by Kang et al. [44], this was an average of 11.7% per day.”

Synthesize the data presented in the above paragraph instead of presenting each study separately.

Overall in the discussion there is a large amount of information presented that would have been meaningful incorporated into the Introduction. This information from references would make a great Introduction to the issues.

What are the limitations of using accelerometer data for young children? And the use of a single accelerometer instead of using multiples? Might there be limitations with the use of a single accelerometer? Add this information to your limitations

Conclusion

You present that lower education levels are associated with the lower physical activity. Could the real impact be that lower education parents work at unskilled or low skilled work and therefore are physically exhausted when they arrive home from work. Or is the implication that low education parents don’t know that physical activity is important?
