# Supplementary material for: A comparative analysis of objectively assessed physical activity levels in kindergarten and home among children aged 5 to 6
Source: PLoS One. 2026 May 15;21(5):e0349582. doi: 10.1371/journal.pone.0349582 (PMC13178922; doi:10.1371/journal.pone.0349582)
Supplement: Attachment — Submitted filename: R1 REPORT-PONE-D-25-56543.docx [file pone.0349582.s003.docx]

**I still have the following methodological concerns:**

1. The authors seem to have fixed some major methodological concerns that I raised by simply listing them as limitations rather than providing more robust analysis or stronger data. The limitations section should not be used as a safety net in methods. For example;

- By failing to provide segmentation accuracy, we miss a solid way to prove exactly when a child was at school versus at home. Consider to either re-analyze the data or provide a more objective validation. While it’s good to be transparent, simply admitting a flaw exists doesn't make the data any more reliable.
- The failure to have separate criteria for a valid kindergarten day and a valid home day means the rigor of the data for each specific setting remains unquantified. Do you still want to qualify/retain this as a limitation?

2. The study continues to report sex (boys vs. girls) and setting (home vs. school) as independent factors without statistically testing how they interact. Are you able to carry out a formal interaction analysis instead of acknowledging this as a study limitation? For instance, do boys get a much bigger boost in activity when they get home compared to girls? Or does the kindergarten environment suppress activity in girls more than it does in boys? The paper has provided the "what," but skipped the statistical test that explains the "how."
3. Without setting-specific wear criteria, it remains unclear if a child's home physical activity is being calculated based on 2 hours or 6 hours of valid data. This raises an issue of data quality.
