# Supplementary material for: A comparative analysis of objectively assessed physical activity levels in kindergarten and home among children aged 5 to 6
Source: PLoS One. 2026 May 15;21(5):e0349582. doi: 10.1371/journal.pone.0349582 (PMC13178922; doi:10.1371/journal.pone.0349582)
Supplement: Attachment — Submitted filename: Response_to_Reviewers_auresp_2.docx [file pone.0349582.s004.docx]

**I still have the following methodological concerns:**

1. The authors seem to have fixed some major methodological concerns that I raised by simply listing them as limitations rather than providing more robust analysis or stronger data. The limitations section should not be used as a safety net in methods. For example;

- By failing to provide segmentation accuracy, we miss a solid way to prove exactly when a child was at school versus at home. Consider to either re-analyze the data or provide a more objective validation. While it’s good to be transparent, simply admitting a flaw exists doesn't make the data any more reliable.

**Response:**

We thank the Reviewer for this important comment. We fully agree that objective validation of time segmentation between kindergarten and home would strengthen the methodological rigor of the study.

In the present study, parents were instructed to follow standardized daily schedules, with kindergarten attendance occurring between 8:00 a.m. and 2:00 p.m., and home time thereafter until approximately 8:00 p.m. These time frames were consistent across participating institutions and were clearly communicated to parents prior to data collection. Although parents did not maintain detailed logs documenting exact drop-off and pick-up times, they confirmed adherence to these schedules.

We acknowledge that we were unable to objectively verify the exact timing of transitions between settings (e.g., via activity logs or direct observation). Therefore, we are not able to provide a more precise validation of segmentation accuracy or to re-analyze the data using objectively confirmed time stamps. For this reason, this issue has been retained as a methodological limitation.

However, the potential impact of this limitation is mitigated by the high overall data quality. The average daily wear time was approximately 12.9 hours per participant, indicating near-continuous monitoring, and the total recorded wear time was almost equally distributed between kindergarten and home settings. This suggests that both environments were well represented in the dataset. Consequently, although minor misclassification of time segments cannot be excluded, it is unlikely to have materially affected the observed differences between settings.

- The failure to have separate criteria for a valid kindergarten day and a valid home day means the rigor of the data for each specific setting remains unquantified. Do you still want to qualify/retain this as a limitation?

**Response:** We thank the Reviewer for this comment. We acknowledge that separate wear-time criteria for kindergarten and home periods were not defined, which limits the ability to quantify data rigor at the setting-specific level. As such, we retain this as a methodological limitation.

We would like to clarify that parents did not maintain detailed logs of drop-off and pick-up times. Therefore, even if separate criteria for valid kindergarten and home periods had been defined, it would not have been possible to apply them reliably at the individual level.

Importantly, overall wear time was high (~12.9 hours/day) and evenly distributed between kindergarten and home settings, suggesting balanced data coverage across both environments. Thus, although setting-specific validity thresholds were not applied, the risk of substantial bias due to unequal data representation is likely limited.

2. The study continues to report sex (boys vs. girls) and setting (home vs. school) as independent factors without statistically testing how they interact. Are you able to carry out a formal interaction analysis instead of acknowledging this as a study limitation? For instance, do boys get a much bigger boost in activity when they get home compared to girls? Or does the kindergarten environment suppress activity in girls more than it does in boys? The paper has provided the "what," but skipped the statistical test that explains the "how."

**Response:** We thank the Reviewer for this valuable comment. In line with this suggestion, we have conducted an additional analysis to formally test the interaction between setting (kindergarten vs. home) and sex. The results of this interaction analysis have now been included in the Results section.

3. Without setting-specific wear criteria, it remains unclear if a child's home physical activity is being calculated based on 2 hours or 6 hours of valid data. This raises an issue of data quality.

**Response:** We thank the Reviewer for this comment. We acknowledge that, in the absence of setting-specific wear-time criteria, the exact amount of valid data contributing to home and kindergarten periods at the individual level cannot be precisely quantified. This has been recognized as a limitation.

However, we would like to emphasize that overall wear time was high (~12.9 hours/day), indicating near-continuous monitoring, and the total recorded wear time was almost equally distributed between kindergarten and home settings. This suggests that both environments were similarly and adequately represented in the dataset. Therefore, although variability at the individual level cannot be fully excluded, it is unlikely that the results are driven by substantial imbalances in data coverage across settings.
